# Deep learning-based image analysis methods for brightfield-acquired multiplex immunohistochemistry images

**DOI:** 10.1186/s13000-020-01003-0

**Published:** 2020-07-28

**Authors:** Danielle J. Fassler, Shahira Abousamra, Rajarsi Gupta, Chao Chen, Maozheng Zhao, David Paredes, Syeda Areeha Batool, Beatrice S. Knudsen, Luisa Escobar-Hoyos, Kenneth R. Shroyer, Dimitris Samaras, Tahsin Kurc, Joel Saltz

**Affiliations:** 1 Department of Pathology, Stony Brook University Renaissance School of Medicine, 101 Nicolls Rd, Stony Brook, 11794 USA; 2 Department of Computer Science, Stony Brook University, 100 Nicolls Rd, Stony Brook, 11794 USA; 3 Department of Biomedical Informatics, Stony Brook University Renaissance School of Medicine, 101 Nicolls Rd, Stony Brook, 11794 USA; 4 Department of Pathology, University of Utah, 2000 Circle of Hope, Salt Lake City, UT 84112 USA; 5 Department Therapeutic Radiology, Yale University, 15 York Street, New Haven, CT 06513 USA

**Keywords:** Multiplex immunohistochemistry, Digital pathology image analysis, Deep learning, Tumor immune microenvironment

## Abstract

**Background:**

Multiplex immunohistochemistry (mIHC) permits the labeling of six or more distinct cell types within a single histologic tissue section. The classification of each cell type requires detection of uniquely colored chromogens localized to cells expressing biomarkers of interest. The most comprehensive and reproducible method to evaluate such slides is to employ digital pathology and image analysis pipelines to whole-slide images (WSIs). Our suite of deep learning tools quantitatively evaluates the expression of six biomarkers in mIHC WSIs. These methods address the current lack of readily available methods to evaluate more than four biomarkers and circumvent the need for specialized instrumentation to spectrally separate different colors. The use case application for our methods is a study that investigates tumor immune interactions in pancreatic ductal adenocarcinoma (PDAC) with a customized mIHC panel.

**Methods:**

Six different colored chromogens were utilized to label T-cells (CD3, CD4, CD8), B-cells (CD20), macrophages (CD16), and tumor cells (K17) in formalin-fixed paraffin-embedded (FFPE) PDAC tissue sections. We leveraged pathologist annotations to develop complementary deep learning-based methods: (1) *ColorAE* is a deep autoencoder which segments stained objects based on color; (2) *U-Net* is a convolutional neural network (CNN) trained to segment cells based on color, texture and shape; and (3) ensemble methods that employ both *ColorAE* and *U-Net*, collectively referred to as *ColorAE:U-Net*. We assessed the performance of our methods using: structural similarity and DICE score to evaluate segmentation results of ColorAE against traditional color deconvolution; F1 score, sensitivity, positive predictive value, and DICE score to evaluate the predictions from ColorAE, U-Net, and ColorAE:U-Net ensemble methods against pathologist-generated ground truth. We then used prediction results for spatial analysis (nearest neighbor).

**Results:**

We observed that (1) the performance of ColorAE is comparable to traditional color deconvolution for single-stain IHC images (note: traditional color deconvolution cannot be used for mIHC); (2) ColorAE and U-Net are complementary methods that detect six different classes of cells with comparable performance; (3) combinations of ColorAE and U-Net in ensemble methods outperform ColorAE and U-Net alone; and (4) ColorAE:U-Net ensemble methods can be employed for detailed analysis of the tumor microenvironment (TME).

**Summary:**

We developed a suite of scalable deep learning methods to analyze 6 distinctly labeled cell populations in mIHC WSIs. We evaluated our methods and found that they reliably detected and classified cells in the PDAC tumor microenvironment. We also utilized the ColorAE:U-Net ensemble method to analyze 3 mIHC WSIs with nearest neighbor spatial analysis. We demonstrate a proof of concept that these methods can be employed to quantitatively describe the spatial distribution of immune cells within the tumor microenvironment. These complementary deep learning methods are readily deployable for use in clinical research studies.

## Background

Multiplex IHC (mIHC) and multiplex immunofluorescence (mIF) are methods that are used to detect multiple targets in a single histologic section with different colored chromogens (e.g. DAB, AES, TMB, BCIP) or fluorophores for mIHC and mIF, respectively. Traditional IHC employs a single antibody for each tissue section, where multiple markers are assessed in consecutive serial tissue sections. Therefore, mIHC increases our ability to observe direct interactions between cells within the appropriate histological context in a single tissue section and maximizes the number of markers that can be assessed with limited tissue. Fully automated mIHC and mIF platforms are being deployed as high-throughput assays for future use in CLIA/CAP certified laboratory settings.

We used a mIHC platform to visualize inflammatory responses in the tumor microenvironment of pancreatic ductal adenocarcinoma (PDAC). We chose this model system since PDAC is one of the deadliest types of cancer, known to be poorly immunogenic and unresponsive to currently available immunotherapeutic treatment options [1, 2]. Investigation of the relationship between PDAC and the inflammatory microenvironment could be further advanced by the development of methods that quantify cell populations and their distribution within the tumor microenvironment in an automated and reproducible fashion. We utilized mIHC rather than mIF due to the decay of fluorophores over time, challenges associated with interpreting mIF from the lack of histologic context, and need for specialized fluorescence or spectral imaging instrumentation that is labor intensive, expensive, and requires expertise [3–20].

The analysis of inflammatory responses in the tumor microenvironment (TME) is increasingly significant as the development and deployment of immunotherapeutic protocols continues to increase for many types of cancer [2, 16, 18, 21–30]. Investigations of tumor-immune interactions in the TME using mIHC may help improve clinical outcomes through the discovery of predictive and prognostic biomarkers [14, 26, 27, 30–64]. Since tumor-immune interactions are exquisitely complex and diverse across different types and subtypes of cancer, meaningful analysis of the TME requires the detection and classification tumor cells and immune cell subtypes to (1) characterize the functional immune status of the TME, (2) identify potential intrinsic immune biomarkers, and (3) provide insight into the expression of known immunotherapeutic drug targets. In order to clinically implement mIHC, pathologists have to be able to meaningful interpret multicolored tissue sections that contain several types of labeled cells.

Thus, computational methods are being explored to augment traditional histologic examination in an effort to help reliably detect and classify multiple distinct cell populations in digital whole slide images (WSIs) of mIHC-stained tissue sections [4, 6, 7, 9, 11, 13–15, 19, 65–67]. We developed a suite of algorithms that leverage deep learning to overcome the need to use specialized multispectral imaging instrumentation for quantitative analysis of mIHC WSIs containing six or more distinctly colored chromogens. Our methods utilize computationally inexpensive deep learning convolutional neural networks (CNNs) that are trained to separate colors and classify cells in a time efficient and comprehensive manner with limited training data. The success of each of the methods demonstrates the value of using deep learning-based image analysis methods for automated analysis of mIHC WSIs. Therefore, we also present an application of our methods to quantitatively describe the spatial relationships between tumor and immune cells in PDAC as an example of the types of insights that can be gained from such analysis.

We report our efforts to develop and test complementary color deconvolution and immune cell classification methods by using deep learning CNNs. We developed a suite of deep learning tools with two distinct algorithmic approaches and combinations of these methods. Our suite of deep learning tools includes (1) a deep autoencoder for color decomposition, (2) a U-Net based approach for cell segmentation, and (3) multiple ensemble approaches intended to increase the positive predictive value (PPV) of cell detection and classification. This manuscript reports the development of these methods in a specific use case to quantitatively analyze the expression of six biomarkers to study tumor immune interactions in PDAC. Our goal was to develop these methods to build robust and scalable analytic pipelines that can be easily configured and deployed to analyze mIHC WSIs for a wide array of research and clinical applications.

## Methods

The identification of different types of cells with mIHC is based on the unique colors of chromogenic agents that are localized to specific cells in formalin-fixed and paraffin-embedded (FFPE) tissue sections. However, available research and commercial software used for color decomposition is typically limited to three or four channels. Therefore, we developed multiple deep learning models with training data for six different colored chromogens in a PDAC mIHC panel. Our suite of deep learning tools included: (1) ColorAE, (2) U-Net, and (3) ColorAE:U-Net ensemble models to detect color-labeled immune cell types and tumor cells in mIHC WSIs without the need for spectral deconvolution during image acquisition by digital slide scanners.

### Tissue specimens

FFPE tissue sections (5 μm thickness) from ten cases of PDAC, provided by the archival collections of the Department of Pathology at Stony Brook University Hospital, were obtained for this mIHC pilot study. These cases represent a subset of cases from a previously published cohort [68]. Tumor sections were reviewed from each case to identify the tissue block with the greatest area of viable tumor and those with < 1 cm^2^ of tumor were excluded. The tissue slides were de-identified and multiple security measures, including password protection and storage of the password key on a computer without network access were used to ensure that no patient identifiers could be accessed. All of the members of our research team members have undergone CITI human subjects and medical ethics training.

### Multiplex IHC

**Fig. 1:**
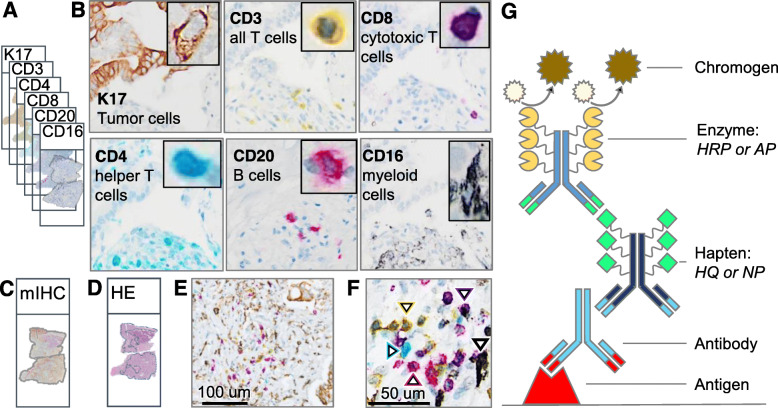
IHC markers in the PDAC mIHC panel to study tumor immune interactions. **A**. Traditional IHC was used to stain six adjacent serial tissue sections with one biomarker per tissue section. Each biomarker is designated by a specific color. **B**. Representative images from single-marker IHC in serial tissue sections showing expression in the same region of interest (except for examples of sparsely distributed B-cells from a different area in the corresponding histologic section). Each inset shows the cellular expression of the corresponding marker at higher magnification. **C**. Multiplex IHC (mIHC) in adjacent serial section. **D**. Hematoxylin and eosin (H&E) in adjacent serial sections with delineation of the tumor region by a pathologist. **E**. mIHC with six IHC markers on a single tissue section with PDAC. **F**. At the highest magnification, each of the five immune cell classes are labeled with a white arrowhead outlined in the color corresponding to chromophore color. T-cell subtypes include CD3+ (yellow), CD4+ (teal), CD8+ (purple); B-cells denoted by CD20+ (red); and myeloid cells identified by CD16+ (silver-black). **G**. Indirect mIHC using hapten-conjugated secondary antibodies. Primary antibody binds to target antigen; secondary anti-mouse or anti-rabbit IgG antibody conjugated to synthetic haptens (HQ or NP) to bind primary antibody; and anti-hapten tertiary antibody conjugated to multiple enzyme molecules (e.g. horseradish peroxidase (HRP) or alkaline phosphatase (AP)) to bind secondary antibody. Chromogen substrate reacts with enzymes to generate insoluble unique color signal at the site of the targeted antigen

mIHC of the tissue sections was performed at Roche Diagnostics (Tuscon, AZ), using a Discovery Ultra Autostainer (Roche/Ventana, Oro Valley, AZ). Tissue slides were baked at 60 degrees Celsius for 20 min, followed by 3 × 8-min deparaffinization cycles, antigen retrieval in high pH buffer (CC1, Roche/Ventana), and treatment to block endogenous peroxidase (Inhibitor CM, Roche/Ventana). Antibodies for CD3, CD4, CD8, CD16, and CD20 were provided by Roche/Ventana and an antibody to K17, a biomarker of the most aggressive subtype of PDAC [68] was provided by KDx Diagnostics (Los Gatos, CA). mIHC staining was performed using horseradish peroxidase (HRP)- and alkaline phosphatase (AP)-based protocols with different colored chromogens (e.g. Yellow:CD3 T-cells, Teal:CD4 helper T-cells, Purple:CD8 cytotoxic T-cells, Red:CD20 B-cells, Black:CD16 myeloid cells, and brown (DAB):cancer cells) [68–71]. Secondary monoclonal antibodies to rabbit primary antibodies for CD16, K17, CD8, and CD4, and K17 were conjugated to the HQ hapten; monoclonal antibodies to primary rabbit primary antibodies to CD3 and CD20 were conjugated to the NP hapten (Fig. 1G). After each round of staining, antibody complexes were removed using CC2 (Roche/Ventana), a pH 6.0 citrate/acetate-based buffer containing 0.3% SDS, and heating the slide to 93 degrees for 8 min [72]. Details of the mIHC protocol are outlined in supplemental Table 1; a complete list of required reagents including washes and buffers is included in supplemental Table 2.

### Optimization of IHC protocol

Before finalizing the mIHC protocol, we optimized conditions and validated the staining patterns. *Controls for individual antibodies:* Using two PDAC cases, we stained 6 serial sections with individual antibodies that followed the sections cut for mIHC (Fig. 1A-B). We confirmed that the quality of staining, color intensity, and patterns of IHC staining in each single-stained slide matched the pattern produced with the same antibody in the mIHC slide. In addition, we ran negative controls that substituted diluent for each of the primary antibodies and secondary antibodies. *Heat denaturation controls:* Sensitivity of the antigens to repeated denaturation steps was evaluated in adjacent tissue sections prior to application of the primary antibody. Antigens that were sensitive to repeated denaturation were placed earlier in the sequence.

### Image capture and preparation

After mIHC tissue sections were completed, an Olympus VS120 microscope (Olympus, Tokyo, Japan) was used to scan glass slides and generate digital WSIs at 40x magnification with a resolution of 0.175 μm per pixel. WSIs were partitioned into patches in order to obtain training data to develop two distinct deep learning models to detect, classify, and segment distinct types of cells in the mIHC WSIs. We selected two cases with abundant tissue and obtained six additional serial sections for individually staining with each of the markers in the PDAC mIHC panel for further validation studies.

### Generation of ground truth data

**Fig. 2:**
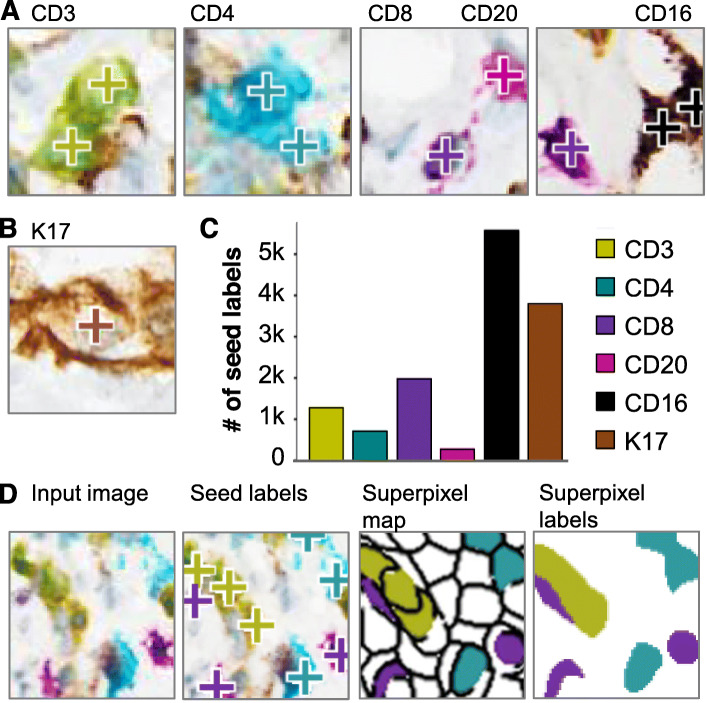
Annotation of patches with seed labels and generation of per-pixel training data. **A**. Examples of CD3+, CD4+, CD8+ and CD20+ lymphocytes, CD16+ myeloid cells and **B**. K17+ PDAC tumor cells with seed labels overlaid (+). **C**. Number of seed labels for each cell class, across all patches used for training. **D**. Input image; input image with seed labels overlaid; superpixel map generated based on the input image with superpixels containing different seed labels colored accordingly; and the superpixel labels used to train the models (based on seed labels and superpixel map)

A set of 80 patches (1920 × 1200 pixels) were selected from representative high-density tumor regions from 10 mIHC WSIs. Six cases were used to generate the training dataset (10 patches per case); four separate cases were selected for the test set (5 patches per case). Since manually delineating the boundaries of individual cells to provide per-pixel annotations is time and cost prohibitive, we utilized seed labels and superpixels (Fig. 2A,B,D) to create a relatively large training data set of per-pixel annotations (superpixel labels, Fig. 2D). A pathologist examined each patch and placed a seed annotation at the center of each cell to indicate the identity of the cell based on staining. This seed label corresponded to the dominant stain across the cell.

Superpixel computation is a well-developed technique in computer vision [73]. The superpixel method works by partitioning an image into small regions called superpixels, where color is relatively homogeneous within each superpixel (Fig. 2D). Each superpixel containing a seed label is assigned the corresponding label; the remaining superpixels are considered background pixels (Fig. 2D). The resulting superpixel annotations are called super-pixel labels (Fig. 2D). Even though the superpixel label may not exactly match the boundaries of the cells, we were able to improve the strength of the annotations to train the models without increasing the labor needed to generate the labels.

### ColorAE

**Fig. 3:**
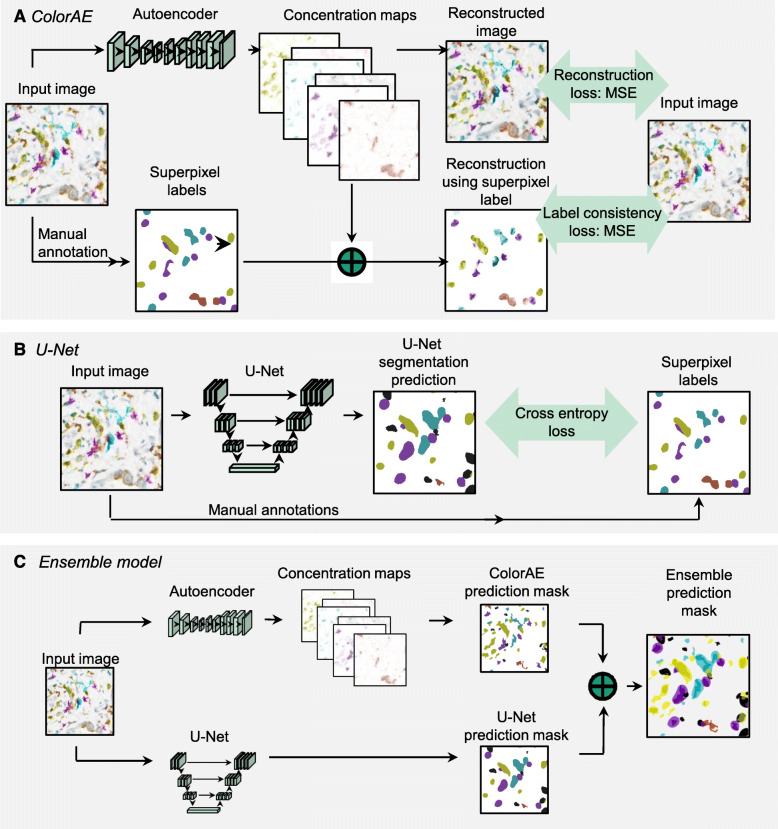
Algorithm training. **A**. *ColorAE training*. Input image is run through an autoencoder to yield concentration maps of each color (6 distinct mIHC stain colors: yellow, teal, purple, red, black, brown; blue hematoxylin nuclear counterstain; and background.) Two loss functions are applied to ensure that the reconstructed image has the highest fidelity to the original image and expert weak annotations. **B**. *U-Net* *training*. Input image was run through a U-Net. Cross entropy loss function was applied to maximize fidelity to superpixel labels derived from manual annotation of the input image. **C**. *Ensemble method* workflow. Input image is run through the autoencoder and U-Net to generate predictions as shown above

The color in any given pixel in mIHC WSIs is combination of primary colors. ColorAE predicts the proportion of different colors corresponding to different stains and referred to as color concentration for each pixel (Fig. 3A). By the Beer Lambert Law [74], the summation of the colors of different stains, weighted by their concentrations, is equal to the observed color. This linear relationship is true only after the colors are mapped into optical densities, i.e., the negative logs of the colors after normalization. This provides a means to recover the color concentrations for every pixel when three or fewer colored stains are used by directly solving the linear equation system [75]. If there are more than three stains, the linear equation system becomes underdetermined. Even though one may use more advanced techniques including sparsity regularization and deep neural networks [76–80], these methods do not capture the rich amount of information from colored stains between adjacent pixels, especially for our mIHC images with up to 6 stains.

Our proposed method, ColorAE, is an autoencoder that fully exploits the rich spatial information between stains of adjacent pixels. We briefly introduce the method and published the technical details in parallel (Fig. 3A) [75]. An autoencoder is a deep neural network that applies multiple layers of convolutions to the input image so that it is converted to a low resolution, high dimensional latent space representation (Fig. 3A). A series of deconvolutions are then applied to this latent representation to recover an output of the same resolution of the original image (Fig. 3A). In our case, the neural network is trained to predict an 8-channel image of the same size as the input image. The 8 channels correspond to the concentration maps of six IHC stains, hematoxylin (blue), and background (grey) values (Fig. 3A). More details about the network architecture can be found in [81].

To train the ColorAE model, we introduce two loss functions. A reconstruction loss compares the reconstructed image with the input image pixel-by-pixel by using a mean squared error (MSE) (Fig. 3A). This loss alone is insufficient; due to the excessive number of colored stains, multiple different color decompositions can provide the same reconstruction. In order to find the optimal decomposition solution, we leveraged weak-form supervision from human annotators through a label consistency loss function. We created another reconstructed image by using only the superpixel label colored stain and the color concentration value at each pixel (Fig. 3A). By requiring the reconstructed image be as close to the original image, the superpixel label for each pixel is designated as the dominant colored stain.

### U-net

**Fig. 4:**
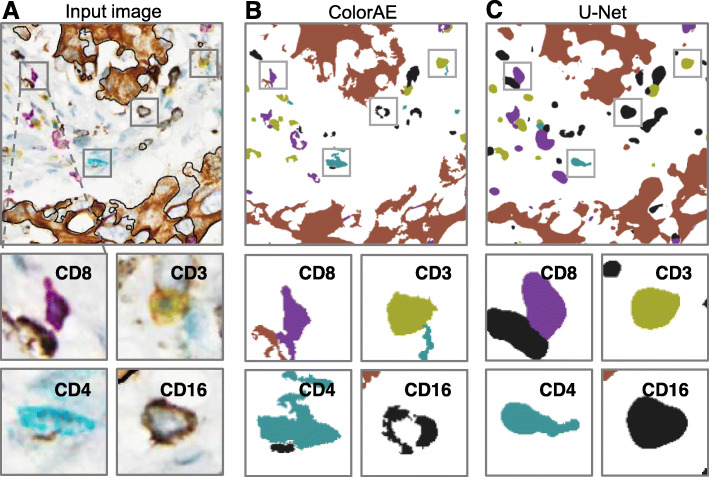
ColorAE and U-Net predictions. **A**. mIHC input image of tumor microenvironment, with a representative cell from each class of immune cells (magnified below). **B**-**C**. ColorAE and U-Net prediction masks based on the original image

The second method is a segmentation CNN based on the U-Net architecture [81]. The U-Net architecture is specifically characterized by the skip connections between the encoding and decoding path and has proven efficient in various medical image segmentation tasks (Fig. 3B). Similar to ColorAE, this model also uses the superpixel labels. U-Net is trained by minimizing the cross-entropy loss. In particular, the U-Net model is trained with dropout and weighted cross entropy loss in order to account for the class imbalance in the training data and learns to segment the different cell classes. It is different from ColorAE in that it does not try to reconstruct the input image or generate stain concentration maps. U-Net is trained to generate features that differentiate the different cell classes according to the provided labels. In that sense it is less constrained than ColorAE. The resulting segmentation maps are not as fine detailed as ColorAE but prove to provide complementary information as seen visually in Fig. 4 and through evaluation of the ensemble methods.

### Ensemble methods

The ColorAE:U-Net ensemble methods combine the respective strengths of the ColorAE and U-Net deep learning methods (Fig. 3C). Color deconvolution with ColorAE was designed to recover the color composition of an image by predicting the color composition for each pixel, whereas U-Net [81] identifies different types of cells without performing overt color deconvolution. We describe the four ensemble methods below.

**Fig. 5:**
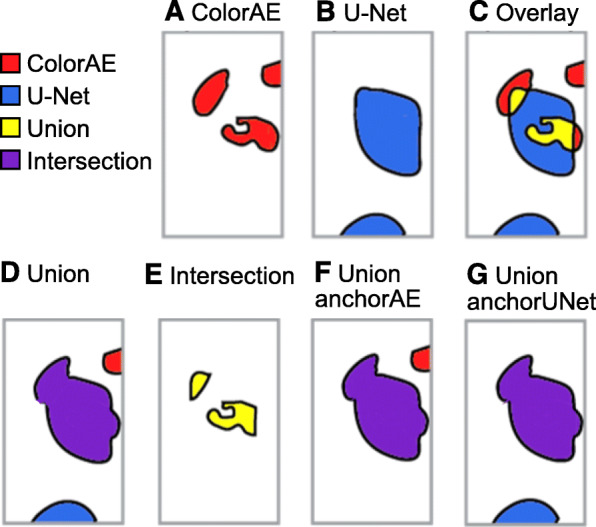
ColorAE, U-Net, and ensemble predictions. All panels show predictions from the same cell class from the same input image. *Top panel*: **A**-**C**. ColorAE and U-Net predictions are shown for a single cell class are shown individually and overlaid. *Bottom panel*, *ensemble methods*: **D**. Union includes predictions from both ColorAE (red) and U-NET (blue): any overlapping predictions (detected by both algorithms) are merged into a single mask (purple). **E**. Intersection includes the pixels detected by both algorithms, while excluding any areas of the mask detected by only one algorithm. **F**. UnionanchorAE includes all masks detected by ColorAE and U-Net masks that intersect a ColorAE mask (union) while excluding U-Net masks that do not intersect a ColorAE mask (only detected by U-Net). **G**. UnionanchorUNet includes all masks detected by U-Net and ColorAE masks that intersect a U-Net mask (union) while excluding ColorAE masks that do not intersect a U-Net mask (only detected by ColorAE)

*Union* combines the predictions from both methods; overlapping predictions are combined into a single mask (Fig. 5A,B,D). *Intersection* only includes pixels identified by both algorithms (Fig. 5E). *Union anchor AE* takes the union of the masks and discards any U-Net predictions that do not overlap with a ColorAE prediction (only keeps the connected components that contain colored pixels from the ColorAE mask) (Fig. 5F). *Union anchor U-Net* takes the union of the masks and discards any ColorAE predictions that do not overlap with a U-Net prediction (only keeps the connected components that contain colored pixels from the U-Net mask) (Fig. 5G).

### Spatial analysis

**Fig. 6:**
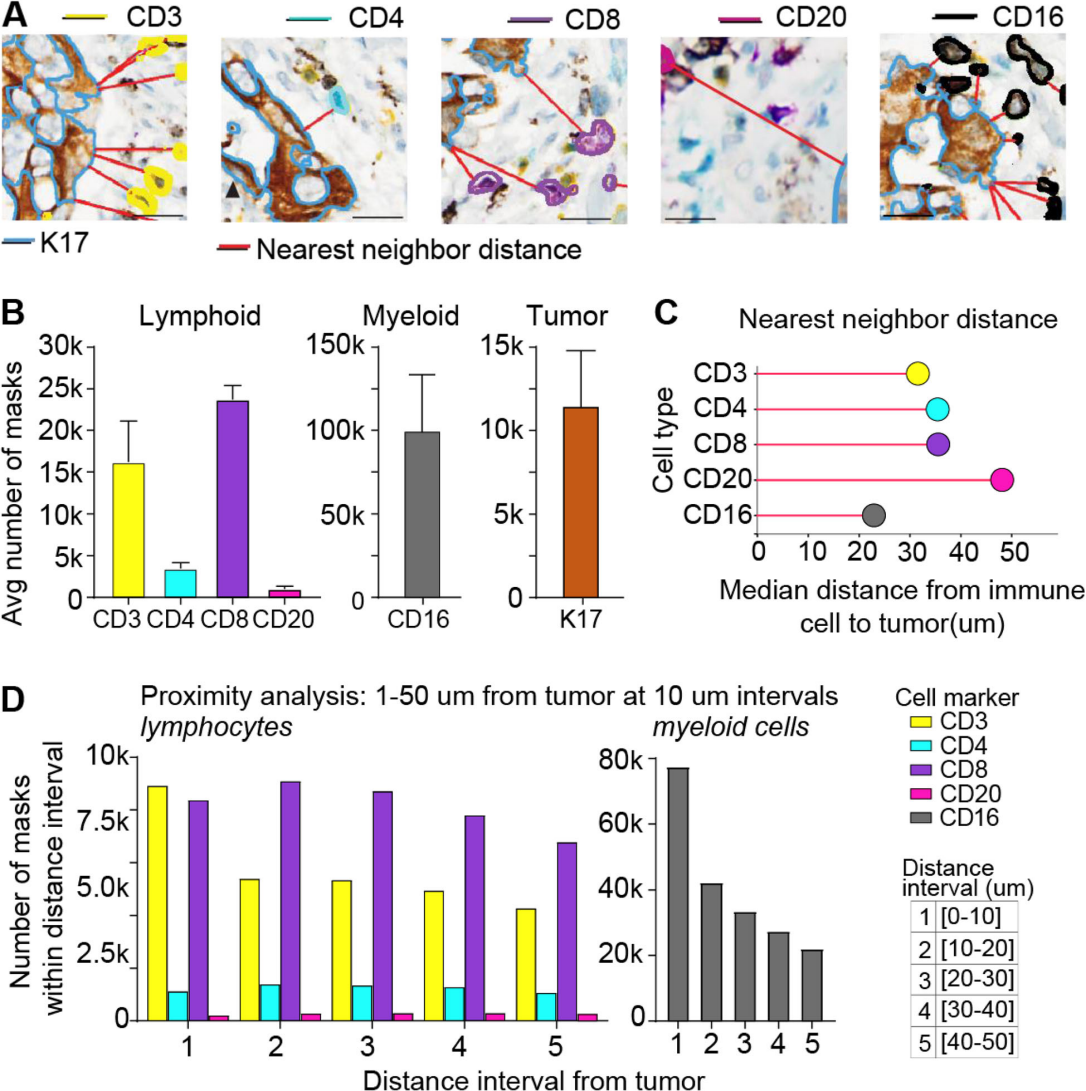
Example of analyses describing immune-tumor spatial relationships in mIHC-stained PDAC tissue. **A**. Representative images of segmentation boundaries of detected tumor nests and immune cells labelled with IHC biomarkers: K17 (blue segmentation boundary), CD3 (yellow boundary), CD4 (teal boundary), CD8 (purple boundary), CD20 (magenta boundary), and CD16 (black boundary). Red lines indicate nearest neighbor distance vector connecting each immune cell to the nearest tumor nest. **B**. Average number of masks per case for each of the different cell classes in three WSIs. **C**. Median nearest neighbor distances for each immune cell class across cases. **D**. Proximity analysis showing the number of detected masks for each cell class at 10 um distance intervals from the tumor boundary. *Note: Nearest Neighbor analyses are asymmetric and Nearest Neighbor analyses from each tumor nest to nearest immune cell are shown in supplemental Fig. 4

We used the results from the best performing algorithm (union anchor AE) for downstream spatial analysis as proof of concept that our algorithm could be employed to survey relationships between cells in the tumor microenvironment. Nearest neighbor distance (NNDist) is determined by the Euclidean length of the shortest vector connecting the edge of one mask or cell (e.g. cytotoxic T-cell) and the edge of the next closest mask or cell (e.g. tumor, as shown in Fig. 6A). In this way, NNDist was calculated with Scipy library to determine the average distance between different types of cells with one and another. Median NNDist distances between immune and tumor cells were found by aggregating them from multiple WSIs and averaging across all of the 2000 × 2000 pixels tiles within the pathologist annotated tumor region (Fig. 1C-D). The pathologist-annotated tumor region was manually annotated based on visual inspection in an adjacent H&E tissue section. Proximity analyses utilized NNDist data and counted the number of immune cells within discrete distance intervals from the nearest tumor mask.

## Results

We evaluated the performance of the proposed methods by using (1) structural similarity and DICE score to compare results from ColorAE to traditional color deconvolution used on a set of consecutive serial tissue sections stained with single colors, (2) F1 score, sensitivity (recall), and positive predictive value (precision) comparing predictions of all algorithms to dilated seed labels, and (3) DICE score to compare predictions of all algorithms with hand-drawn annotations (referred to here as per-pixel labels).

### Evaluation of similarity between ColorAE and color deconvolution approaches on single-stain images

The ColorAE method was explicitly designed to detect different colored stains through classical color decomposition for the single-stained images since they consisted of only three colors [80]. We should note that this method was designed for H&E images and it is limited to the analysis of no more than three colored stains. It cannot be applied to mIHC images. By visual inspection, the results from ColorAE were comparable to those predicted by Vahadane’s method with the notable exception of CD16. This appears to be due to the similarity between black stain and the gray background color and the inability of ColorAE to reliably distinguish between the two in the context of single-stained images (Fig. S1). We utilized the mean structural similarity index (SSim) in 20 randomly selected patches (1440 × 1440 pixels) to quantitatively compare ColorAE and Vahadane’s method. SSim [73] measures the similarity between two predictions with respect to luminance, contrast, and structure over sliding windows. SSim is particularly more robust than traditional methods to measure image prediction quality (e.g. peak signal to noise ratio (PSNR) and mean squared error (MSE)). We also compared masks from the derived segmentation of colored stains, which are computed from automatic determination of color concentration, to the masks from Vahadane’s method in [79] by using the DICE score (Fig. S1, Table 1). The DICE score is computed as twice the area of intersection of the two masks divided by the sum of their individual areas, formally, DSC = 2|A∩B| / (|A| + |B|), where A and B are the regions from the predicted mask and the ground truth mask, respectively.

**Table 1:** Evaluation of ColorAE

Evaluation metrics	CD3	CD4	CD8	CD20	CD16	K17
SSim (color concentration.	0.9	0.87	0.9	0.87	0.54	0.94
DICE (segmentation masks.	0.87	0.81	0.86	0.34	0.59	0.73

### Evaluation of label prediction performance against dilated seed labels

The next set of experiments evaluated the performances of ColorAE, U-Net, and the ensemble methods by comparing the models’ predictions with the ground truth seed labels (Fig. 5). We dilated the seed labels into disks with a diameter of 10.5 um to correspond to the average size of lymphocytes. The masks for each colored stain were evaluated one at a time. Using the predictions and dilated seed labels, we evaluated true positives (TP), false positives (FP), and false negatives (FN). Specifically, TP is the number of connected components in the mask overlapping with the 10 μm disks; FP is the number of connected components in the mask that do not overlap with any disks; and FN is the number of disks that do not overlap with the mask. Since some cell types might be sparse in some patches, we aggregated values for TP, FP, and FN from all of the 20 testing patches. These aggregated values were used to compute standard performance metrics like the F1-score, recall, and precision (Tables 2, 3 and 4).

**Table 2:** F1 Scores evaluating predictions from UNet, ColorAE, and ensemble methods against dilated seed labels

Model	CD3	CD4	CD8	CD20	CD16	K17
ColorAE	0.652	0.728	0.72	0.347	0.796	0.637
UNet	0.628	0.661	0.628	0.353	0.687	0.739
Intersection	**0.698**	0.693	0.722	0.279	0.79	0.749
Union	0.593	0.705	0.628	**0.403**	0.691	0.638
Union anchor AE	0.662	**0.732**	**0.731**	0.346	**0.836**	0.657
Union anchor UNet	0.639	0.674	0.633	0.35	0.7	**0.806**

**Table 3:** Sensitivity (Recall) evaluating predictions from UNet, ColorAE, and ensemble methods against dilated eed labels

Model	CD3	CD4	CD8	CD20	CD16	K17
ColorAE	0.974	0.798	0.868	0.212	0.914	0.871
UNet	0.931	0.675	0.881	0.232	0.991	0.756
Intersection	0.919	0.627	0.865	0.163	0.909	0.727
Union	**0.981**	**0.848**	**0.887**	**0.275**	**0.993**	**0.909**
Union anchor AE	0.974	0.795	0.872	0.212	0.936	0.887
Union anchor UNet	0.933	0.686	0.884	0.229	0.992	0.846

**Table 4:** Positive Predictive Value (Precision) evaluating predictions from UNet, ColorAE, and ensemble methods against dilated seed labels

Model	CD3	CD4	CD8	CD20	CD16	K17
ColorAE	0.49	0.67	0.615	0.945	0.706	0.502
UNet	0.473	0.647	0.488	0.736	0.526	0.722
Intersection	**0.562**	**0.773**	0.619	**0.957**	0.699	**0.771**
Union	0.425	0.603	0.486	0.757	0.53	0.491
Union anchor AE	0.501	0.678	**0.629**	0.944	**0.756**	0.521
Union anchor UNet	0.486	0.663	0.493	0.738	0.541	0.77

### Comparison of a U-net trained with dilated seed labels to U-net trained with superpixel labels

We also compared standard U-Net to the superpixel training labels. Generation of sufficient per-pixel annotations for training is prohibitively expensive. As an alternative to superpixel labels, we dilated the seed labels into disks with a small, conservative, diameter of 2 um and use these masks as training data to train a U-Net. We focused on immune cell markers (CD3, CD4, CD8, CD20) as lymphocytes are relatively regular in size and shape and we did not include CD16 and K17 in this evaluation since the myeloid and tumor cells are irregular in size and shape. The results are reported in supplemental material (Fig. S2, Table S3). We show that U-Net trained with superpixel labels significantly outperforms the baseline U-Net trained on dilated disks.

### Evaluation of label predictions against hand-drawn per-pixel annotations

In the final set of experiments, we generated fine-grained hand-drawn per-pixel segmentation annotations (Fig. S3I-J) in a small test set of 19 patches (470 × 470 pixels) and evaluated performance using the DICE score. The DICE score was used to compare the prediction masks (Fig. S3A-H) with the ground truth mask for each colored stain (Table 5).

**Table 5:** DICE Scores evaluating predictions from UNet, ColorAE, and ensemble methods against per-pixel (hand-drawn) labels

Model	CD3	CD4	CD8	CD20	CD16	K17
ColorAE	**0.649**	0.543	0.764	0.589	0.539	**0.649**
UNet	0.649	0.463	0.676	0.554	0.581	0.649
Intersection	0.457	0.457	**0.769**	0.539	0.530	0.457
Union	0.553	0.540	0.675	**0.599**	0.591	0.553
Union anchor AE	0.611	**0.548**	0.685	0.588	**0.593**	0.611
Union anchor UNet	0.526	0.509	0.676	0.558	0.592	0.526

### Analysis of the PDAC tumor microenvironment

Union anchor U-Net was used to generate predictions for tumor and immune cells throughout the tumor region of three mIHC WSIs (Fig. 6A). We calculated both the number of masks per cell class and area of each mask. Our analyses show that the immune microenvironment is dominated by CD16+ myeloid cells (Fig. 6B, S4A). Nearest neighbor distances were calculated between each immune cell and the nearest tumor mask, minimizing vector length (Fig. 6A). The average myeloid cell was also closer to tumor cells than any lymphoid cell class was. We observed that on average, CD3 + CD4-CD8- T-cells, CD4+ helper T-cells, and CD8+ cytotoxic T-cells were about 11 um further from tumor cells than myeloid cells were, whereas B-cells were 13 um further away than T-cells were (Fig. 6C). The median nearest neighbor distance from tumor to immune cells are 2.9 um for CD16+ myeloid, 23.2 um for CD3 + CD4-CD8- T-cells, 44.5 um for CD4+ helper T-cells, 24.0 for CD8+ cytotoxic T-cells, and 56.9 μm for CD20+ B-cells, respectively (Fig. S4). (Note: Nearest Neighbor analyses are asymmetric and there is a difference between calculating the distances from “immune cells to tumor” versus “tumor to immune cells.” The proximity analysis shows the number of masks (cells of each immune cell class) at increasing distance intervals from tumor cells. Looking at 10 μm intervals starting at the tumor mask boundary, the number of myeloid masks drops significantly with increasing distance from the tumor, in contrast to lymphocytes (Fig. 6D). When looking at 1 μm intervals from 0 to 5 μm from the tumor mask boundary, we see that about 30,000 nearest neighbor myeloid cells were within 1 μm (touching) tumor cells, which dropped to approximately 5000 cells and remained steady for the next 4 intervals (Fig. S4). In comparison, 4400 CD3 + CD4-CD8- T-cells, 165 CD4+ helper T-cells, and 1600 CD8+ cytotoxic T-cells were touching tumor (Fig. S4).

## Discussion

The methods described for image analysis of mIHC-stained slides were designed to be robust, reliable, and easily customizable for future clinical research applications. We developed our suite of analytic methods in an effort to make a clear and significant advancement in the ability to survey the immune landscape of PDAC using deep learning to help unravel the complexity of tumor immune interactions in the TME. Our goals were to develop a scalable suite of methods to analyze PDAC mIHC WSIs in a uniform manner, where we can (1) reliably detect, classify, and enumerate different cell types labeled with different colored biomarkers, (2) calculate the distances between the boundaries of tumor and immune cells in mIHC WSIs, and (3) perform spatial analyses to quantitatively describe a large number of diverse tumor immune interactions in multicolored mIHC WSIs without needing expensive multispectral imaging instrumentation.

Our models leverage CNNs trained with this ground truth data to perform pattern recognition functions with statistical multivariate algorithms to predict color and classify all of the different types of labeled cells in the PDAC mIHC WSIs. The methods described leverage relatively inexpensive seed labels (dots) that can be used to generate training sets. Importantly, the ability to use this form of annotation significantly decreases the effort for pathologists to generate training data since placing seed labels at the center of each cell is kuch quicker than manually segmenting all of the different types of cells by hand. Significantly reduced time, labor, and cost leads to the ability to quickly customize analytic pipelines and improves the scalability of our methods.

After training, our models, which are sophisticated statistical algorithms, iteratively improve by learning additional features in successive cycles. These deep learning models perform non-linear regression in large data sets to make predictions that can be used to quantitatively analyze the features of the uniquely colored cell types in mIHC WSIs. However, evaluating these algorithms in terms of their ability to correctly identify and classify six distinct cell populations with variable spatial distributions simultaneously in mIHC WSIs requires many considerations.

The variability of shapes and sizes of cells along with the variable expression of each of the biomarkers in individual cells within the different labeled cell population leads to formidable challenges for any pathologist and algorithm. Furthermore, subtle differences in staining patterns coupled with overlapping color spectra of the chromogens introduces difficulty in color decomposition from the very beginning. For example, intense yellow and light black can both appear brown. This is further complicated in cases where a cell class may be labeled with more than one biomarker, e.g., localization of yellow and purple within the same cell can appear red. Thus, we need digital pathology and image analysis tools that can accurately distinguish different cell classes based on the variability of color that depends on how each types of cell is labeled with a particular biomarker in WSIs of mIHC tissue sections. Despite the technical challenges, the proposed ColorAE method generates color decomposition results that are generally consistent with Vahadane’s method (as shown in supplemental Fig. 1).

However, ColorAE was designed to analyze mIHC WSIs images with more than three colors. ColorAE performed generally better than U-Net at correctly detecting and classifying multicolored immune cells since ColorAE was able to detect lighter colored immune cells that U-Net failed to detect. We also observed that ColorAE captured fine geometric details that U-Net could not, which is particularly evident when comparing CD8 purple masks. There were also very few B-cells in the tissue sections, which resulted in sample bias, where CD20 red B-cells were often misclassified as CD8 purple T-cells and reflected by the low F1-score. CD16 black myeloid cells and K17 brown PDAC cells were also sometimes difficult to distinguish. Both ColorAE and U-Net sometimes misclassified CD16 black as K17 brown and vice versa. Importantly, this seemed to be related in part to the choice of chromogen, where the combination of the black chromogen coupled with the diffuse staining pattern in subsets of myeloid cells appeared brown to the human eye, which can only be distinguished from K17 brown PDAC cells with morphology.

U-Net outperformed ColorAE to detect and classify K17 brown PDAC cells that were counterstained with hematoxylin. Both U-Net and ColorAE can fail to include cell nuclei in the mask since the algorithms generally classify hematoxylin as part of the background. The nuclei of PDAC cells are large and euchromatic with cytoplasmic K17 staining, so it is likely that the algorithms cannot distinguish the nuclei of tumor cells from the background in this use case. Overall, U-Net generally performs better than ColorAE to identify tumor cells. It is important to note that while the tumor cells (and the total tumor mask area) may be underestimated from the exclusion of some nuclei, the boundaries of tumor nests were preserved. Thus, there was still reliable data on tumor nest locations that could be reliably used for downstream spatial analyses.

Furthermore, we show that the methods are complementary, where U-Net had worse recall than ColorAE to detect tumor, but demonstrated significantly better precision. We also observed that ColorAE predicted very detailed masks but was too sensitive in terms of picking up non-specific and background staining. This can be addressed with post-processing by filtering out predictions that contain objects with areas that are below the threshold of being able to be considered as cells. In comparison, the U-Net model produced reasonably conservative predictions, predicting areas of the cell with high intensity staining. However, cells with irregular extensions and low staining intensity were sometimes not detected (Fig. 4). Overall, U-Net performance was limited by the quantity of superpixel labels for training.

In order to address these issues and limitations, we developed the suite of four ColorAE:U-Net ensemble models to detect *intersections*, where a given pixel is predicted to contain a specific color if the pixel is within both of the ColorAE and U-Net masks, and *unions*, where a pixel is predicted to contain a specific color if the pixel is within either the ColorAE or U-Net masks. We recognize that if each cell class is considered independently, the same pixel may be classified as one class by ColorAE and a different class by U-Net (Fig. 4), so we consider both labels in these scenarios. While sometimes this may be a false positive, in other cases this may be reflective of expression of multiple markers on a single cell (e.g. CD3 + CD4+ cells) that results in compound colors. By treating both of the prediction labels assigned to a given pixel as valid, we can capture this phenomenon to some extent.

Even though the qualitative results from all ColorAE, U-Net, and the ensemble methods are generally acceptable, the Union ensemble demonstrated the best sensitivity (recall), as shown in Table 3. This is to be expected as the Union ensemble considers pixels positive for each color if the colored label is predicted by at least one model. In terms of precision, the Intersection ensemble demonstrated the best overall positive predictive value (precision) as shown in Table 4, whereas the Union anchor AE demonstrated the best overall F1 score is considered as shown in Table 2, even though the F1 scores are not directly applicable as a performance metric due to intrinsic variability in the intensity and staining patterns of biomarkers in cells. Although we report considerable progress in developing methods that measure six or more different colored biomarkers in mIHC WSIs, we have to note that these models were trained with a limited dataset and were trained to achieve reasonably good overall performance.

Our results indicate that (1) there is no single universal method that can be the best across all of the performance metrics to target every one of the colored IHC markers and (2) multiple complementary methods can be utilized in analytic pipelines to improve the overall reliability of using computational analysis for mIHC WSIs. In our current use case, we used these novel methods to evaluate the tumor microenvironment PDAC mIHC WSIs. While our focus was to create and evaluate methods for the accurate automated detection of the immune cells in mIHC WSIs, we wanted to demonstrate the types of downstream analyses that can be done to investigate spatial relationships between cell subsets. The nearest neighbor and proximity analyses are based on the spatial positions of all masks across the entirety of the tumor region from a representative PDAC mIHC WSI. For the sake of providing a concrete example, we demonstrate proof that our methods can be used to comprehensively analyze collections of mIHC WSIs.

We emphasize that these methods are still experimental, being refined, and require further comprehensive testing and validation in additional mIHC studies. For example, we observed that segmentation of the boundaries of large PDAC tumor nuclei were occasionally suboptimal and sometimes not detected based on tumor morphology, overlapping nuclei, and obscured nuclear boundaries from intense staining. Even though this limitation can potentially pose a problem with respect to accurately counting every tumor cell, it may not be a significant issue in terms of downstream analyses, including nearest neighbor spatial analyses, since the overall edges of the tumor nests are accurate enough to determine the center point and perimeters of the masks. Nonetheless, the area of K17 brown staining or the number of pixels belonging to K17 masks can still be calculated in order to provide a reasonable estimate of tumor area.

During the microscopic examination of multicolored PDAC mIHC WSIs, what one commonly observes is a fascinating distribution of classical DAB brown-stained K17+ PDAC cells in close proximity to an abundance of black-silver colored CD16+ myeloid cells (e.g. macrophages) with variably interspersed purple colored CD3 + CD8+ T-cells, teal colored CD3 + CD4+ T-cells, and yellow CD3 + CD4-CD8- T-cells. We also have observed that red colored CD20+ B-cells are usually rare in the immune infiltrate associated with PDAC tumor cells, but present in lymphoid aggregates much further away. After histologic review, we utilized our suite of methods to perform spatial analyses in an effort to evaluate the feasibility of quantitatively describing the immune landscape in our PDAC mIHC study. The spatial analyses show how the TME of these PDACs is rich in myeloid cells with a relative dearth of T-cells and B-cells. We also gained insight into patterns of distribution of the three different populations of T-cells. Interestingly, we observed that a significant proportion of the yellow CD3 + CD4-CD8- T-cells may actually represent NK/T-cells, gamma-delta T-cells, or immature T-cells, which can be used to guide other studies.

We are eager to explore whether increasing the size of the cohort will allow us to determine if these patterns are conserved across different cases of PDAC. Furthermore, we are examining the relationship of the spatial patterns of distributions of their different immune cell types with survival data to identify potential prognostic biomarkers. We are also engaged in ongoing studies that are applying these deep learning analytic methods across a much larger cohort of PDAC mIHC WSIs. Future work will also evaluate the relationships between different types of immune cells beyond tumor immune interactions in an effort to better understand cancer immunology.

## Conclusions

We developed a suite of deep learning tools that can be used to create customizable analytic pipelines to analyze mIHC WSIs with good overall performance in a scalable manner. This suite of tools can be reliably implemented to perform cell detection and classification to explore near limitless combinations of multiple IHC biomarkers in one tissue section. To provide a proof of concept, we presented a novel technique to utilize these computational deep learning analyses to enumerate and characterize the spatial distributions of different types of cells in the TME of selected PDAC mIHC WSIs. Since advances in immunotherapy will likely coincide with increased clinical interest in the functional immune status of TME, we believe that these novel deep learning methods complement the adoption of digital pathology and the potential to deploy mIHC for diagnostic testing of cancer tissue specimens. Thus, we hope that these methods facilitate more widespread adoption of mIHC to support precision medicine and accelerate the discovery of biomarkers that can help predict prognosis and guide treatment.

## Supplementary information

**Additional file 1:** Supplemental material.
