# Publisher Correction to: Deep learning-based image analysis methods for brightfield-acquired multiplex immunohistochemistry images

**DOI:** 10.1186/s13000-020-01021-y

**Published:** 2020-09-24

**Authors:** Danielle J. Fassler, Shahira Abousamra, Rajarsi Gupta, Chao Chen, Maozheng Zhao, David Paredes, Syeda Areeha Batool, Beatrice S. Knudsen, Luisa Escobar-Hoyos, Kenneth R. Shroyer, Dimitris Samaras, Tahsin Kurc, Joel Saltz

**Affiliations:** 1 Department of Pathology, Stony Brook University Renaissance School of Medicine, 101 Nicolls Rd, Stony Brook, 11794 USA; 2 Department of Computer Science, Stony Brook University, 100 Nicolls Rd, Stony Brook, 11794 USA; 3 Department of Biomedical Informatics, Stony Brook University Renaissance School of Medicine, 101 Nicolls Rd, Stony Brook, 11794 USA; 4 Department of Pathology, University of Utah, 2000 Circle of Hope, Salt Lake City, UT 84112 USA; 5 Department Therapeutic Radiology, Yale University, 15 York Street, New Haven, CT 06513 USA

**Publisher Correction to: Diagn Pathol 15, 100 (2020)**

**Fig. 1:**
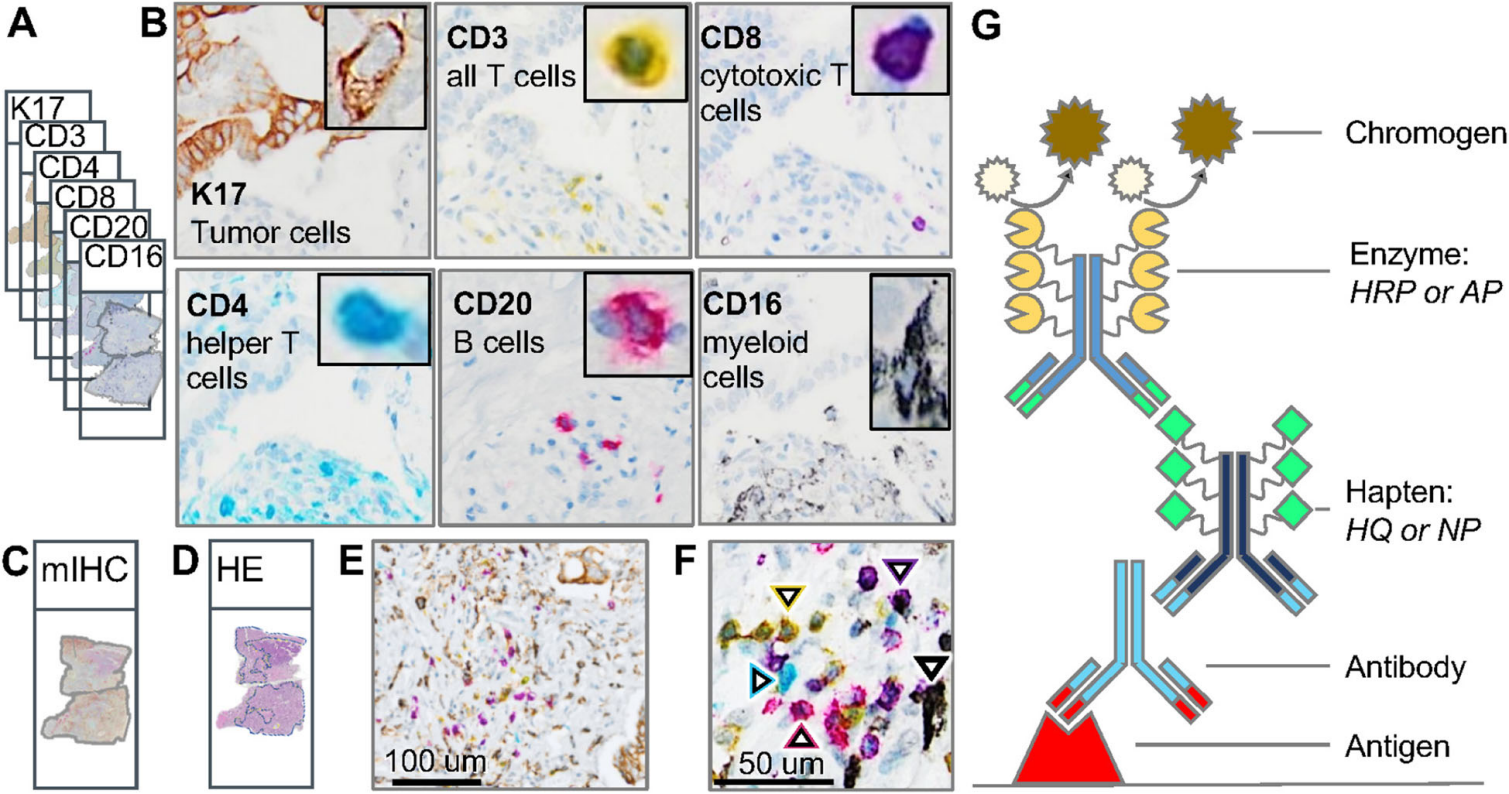
IHC markers in the PDAC mIHC panel to study tumor immune interactions. **a** Traditional IHC to stain six adjacent serial tissue sections with one biomarker per tissue section. Each biomarker is designated by a specific color. **b** Representative images from one marker IHC in serial tissue sections showing expression in the same region of interest (except for examples of sparsely distributed B-cells from a different area in the corresponding histologic section). Each inset shows the cellular expression of the corresponding marker at higher magnification. **C.** Multiplex IHC (mIHC) in adjacent serial section. **d** Hematoxylin and eosin (H&E) in adjacent serial sections with delineation of the tumor region by a pathologist. **e** mIHC with six IHC markers on a single tissue section with PDAC. **f** At the highest magnification, each of the five immune cell classes are labeled with a white arrowhead outlined in the color corresponding to chromophore color. T-cell subtypes include CD3+ (yellow), CD4+ (teal), CD8+ (purple); B-cells denoted by CD20+ (red); and myeloid cells identified by CD16+ (silver-black). **g** Indirect mIHC using hapten-conjugated secondary antibodies. Primary antibody binds to target antigen; secondary anti-mouse or anti-rabbit IgG antibody conjugated to synthetic haptens (HQ or NP) to bind primary antibody; and anti-hapten tertiary antibody conjugated to multiple enzyme molecules (e.g. horseradish peroxidase (HRP) or alkaline phosphatase (AP)) to bind secondary antibody. Chromogen substrate reacts with enzymes to generate insoluble unique color signal at the site of the targeted antigen

**Fig. 2:**
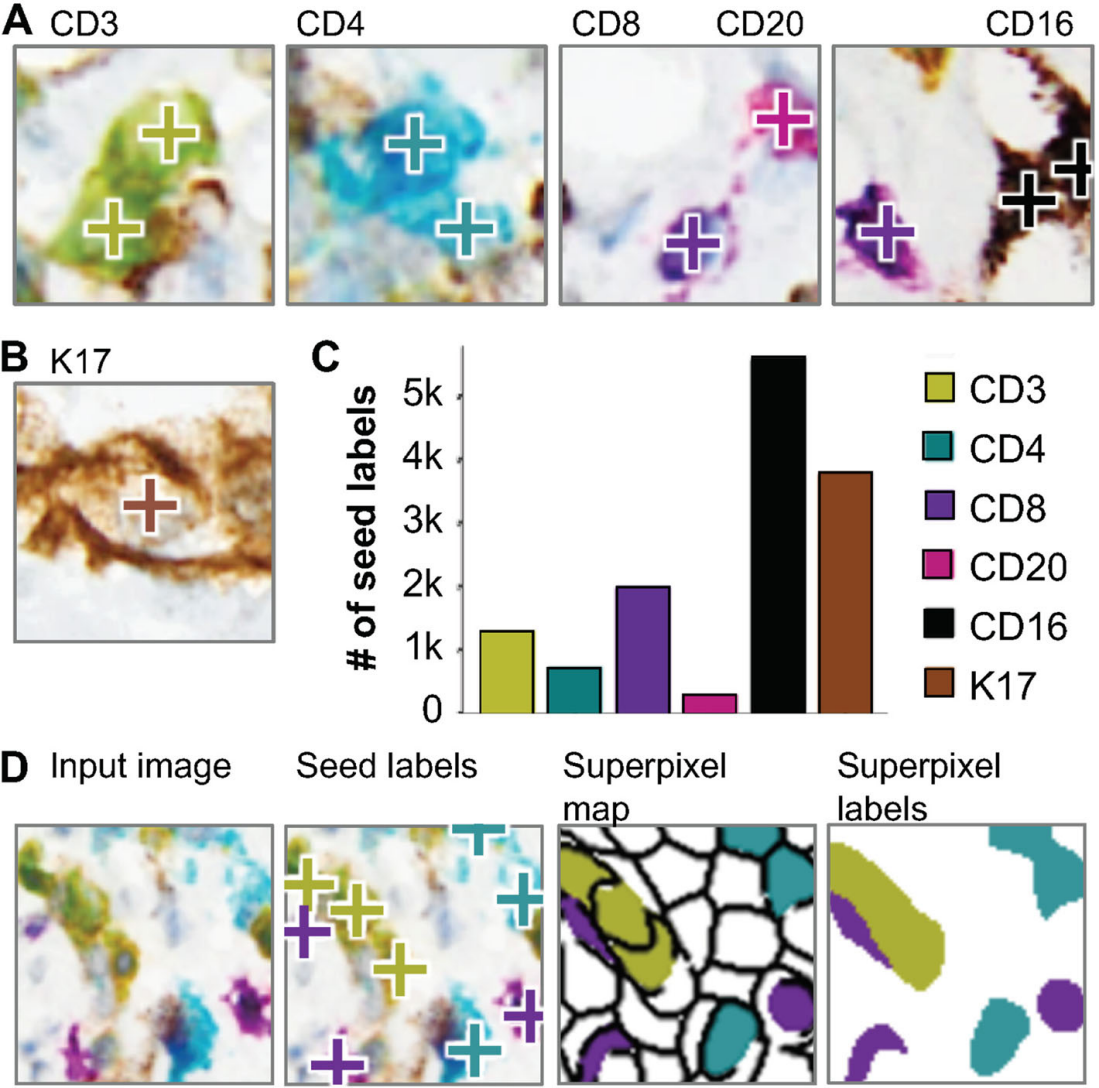
Annotation of patches with seed labels and generation of per-pixel training data. **a** Examples of CD3+, CD4+, CD8+ and CD20+ lymphocytes, CD16+ myeloid cells and **b** K17+ PDAC tumor cells with seed labels overlaid (+). **c** Number of seed labels for each cell class, across all patches used for training. **d** Input image; input image with seed labels overlaid; superpixel map generated based on the input image, with superpixels containing different seed labels colored accordingly; and the superpixel labels used to train the models (based on seed labels and superpixel map)

**Fig. 3:**
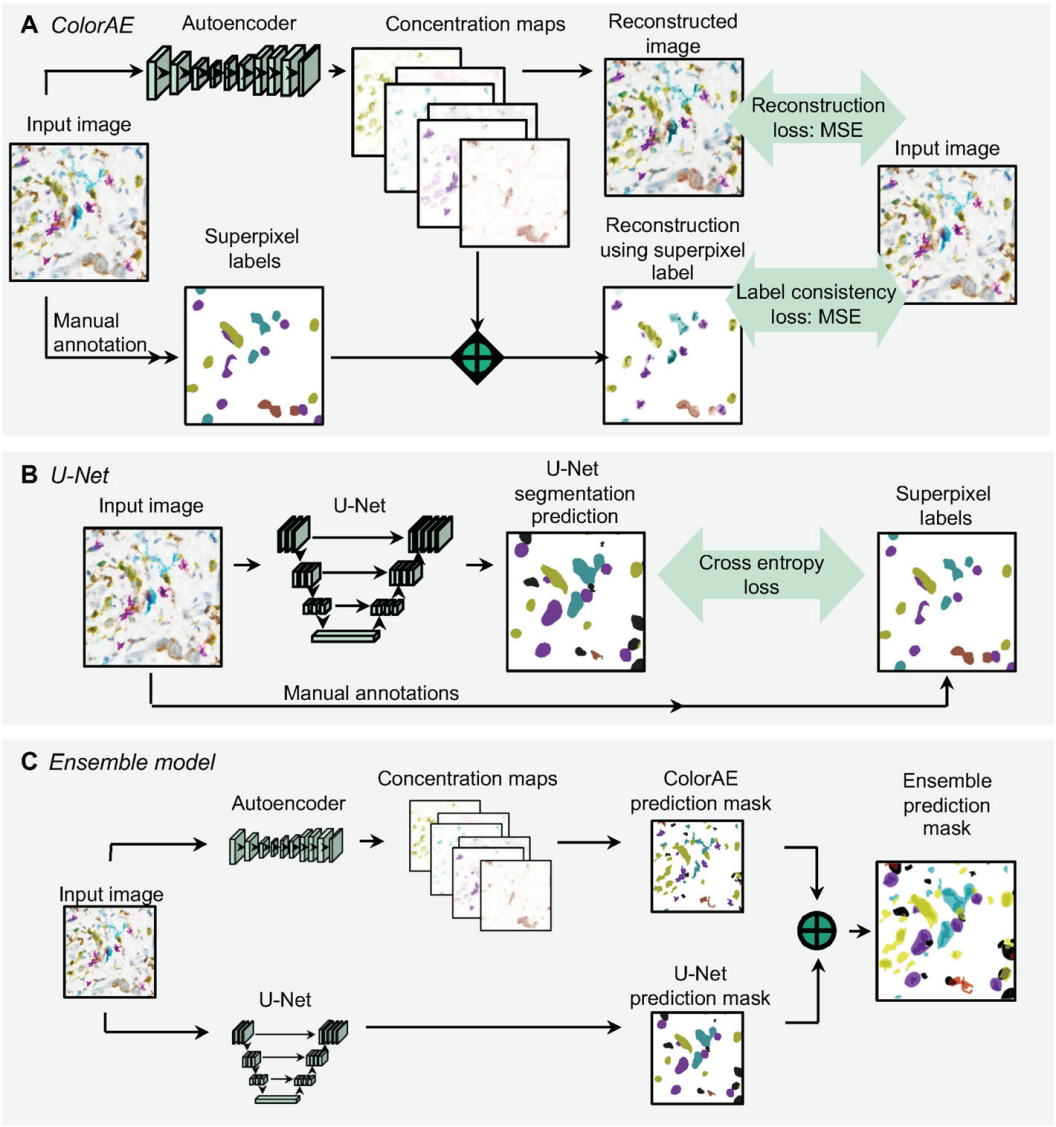
Algorithm training. **a** *ColorAE training.* Input image is run through an autoencoder to yield concentration maps of each color (6 distinct mIHC stain colors: yellow, teal, purple, red, black, brown; blue hematoxylin nuclear counterstain; and background.) Two loss functions are applied to ensure that the reconstructed image has the highest fidelity to the original image and expert weak annotations. **b** *U-Net training*. Input image was run through a U-Net. Cross entropy loss function was applied to maximize fidelity to superpixel labels derived from manual annotation of the input image. **c** *Ensemble method workflow*. Input image is run through the autoencoder and U-Net to generate predictions as shown above

**Fig. 4:**
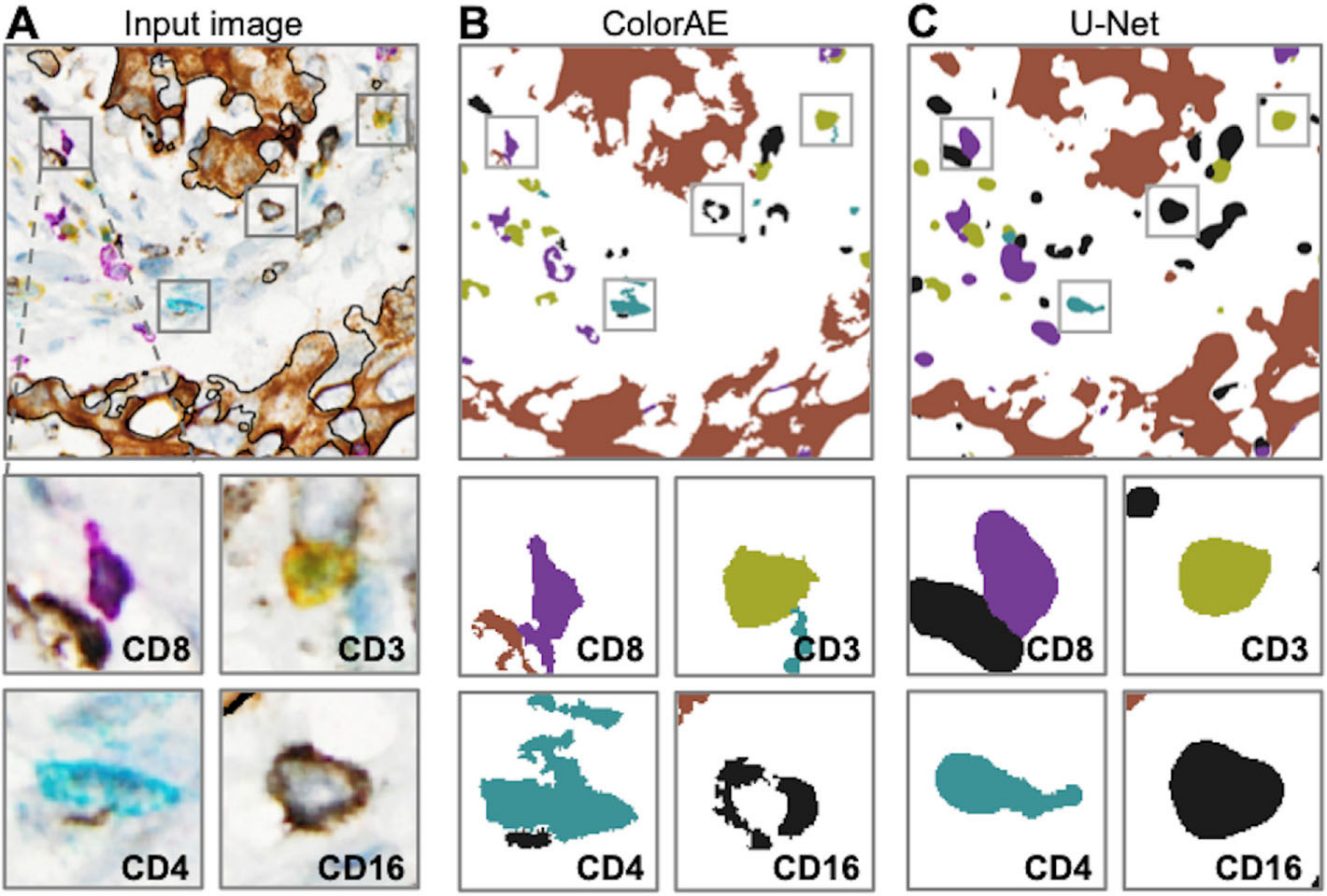
ColorAE and U-Net predictions. **a** mIHC input image of tumor microenvironment, with a representative cell from each class of immune cells (magnified below). **b**-**c** ColorAE and U-Net prediction masks based on the original image

**Fig. 5:**
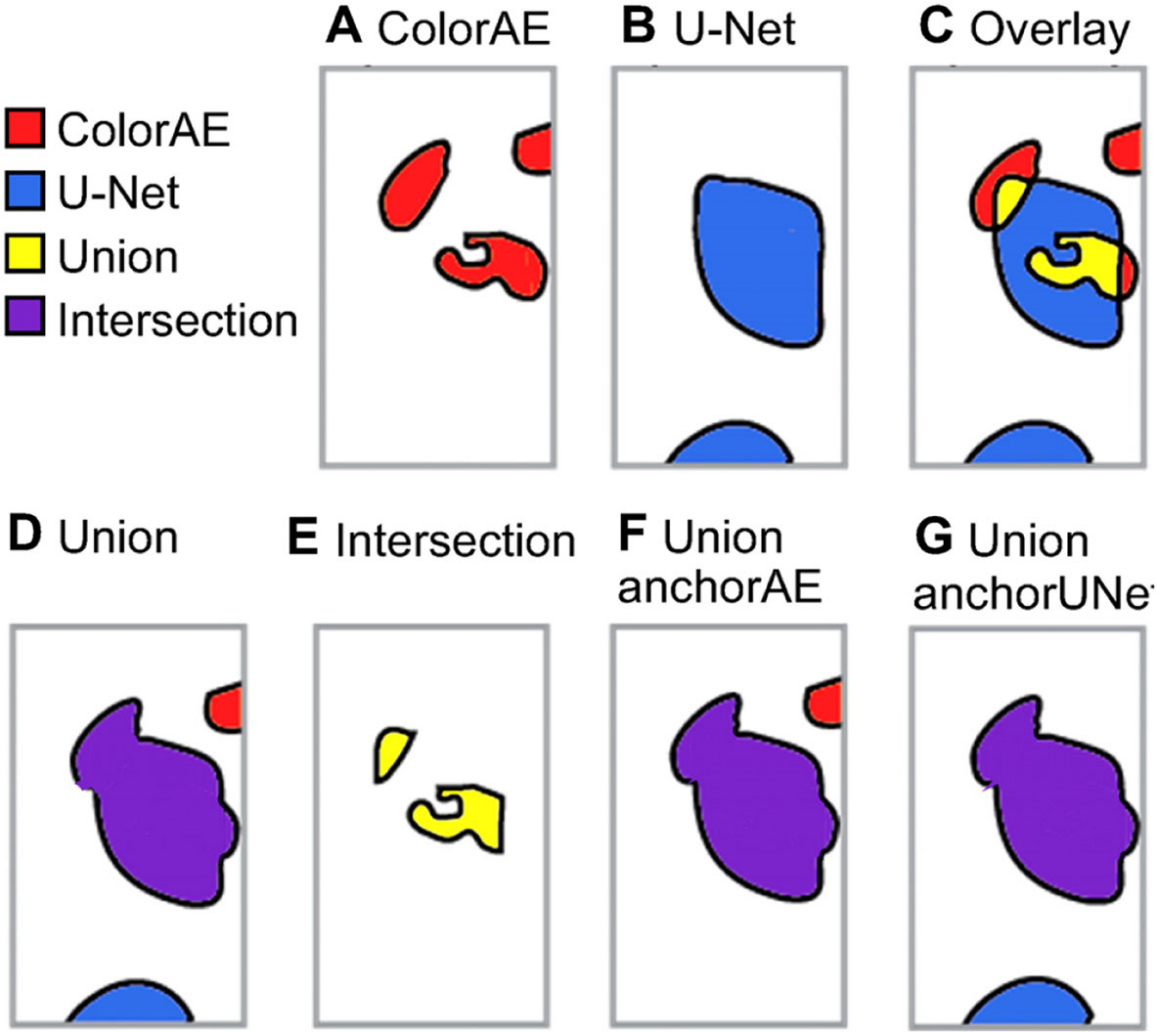
ColorAE, U-Net, and ensemble predictions. All panels show predictions from the same cell class from the same input image. *Top panel:* **a**-**c** ColorAE and U-Net predictions are shown for a single cell class are shown individually and overlaid. *Bottom panel, ensemble methods*: **d** Union includes predictions from both ColorAE (red) and U-NET (blue): any overlapping predictions (detected by both algorithms) are merged into a single mask (purple). **e** Intersection includes the pixels detected by both algorithms, while excluding any areas of the mask detected by only one algorithm. **f** UnionanchorAE includes all masks detected by ColorAE and U-Net masks that intersect a ColorAE mask (union) while excluding U-Net masks that do not intersect a ColorAE mask (only detected by the U-Net). **g** UnionanchorUNet includes all masks detected by U-Net and ColorAE masks that intersect a U-Net mask (union) while excluding ColorAE masks that do not intersect a U-Net mask (only detected by the ColorAE)

**Fig. 6:**
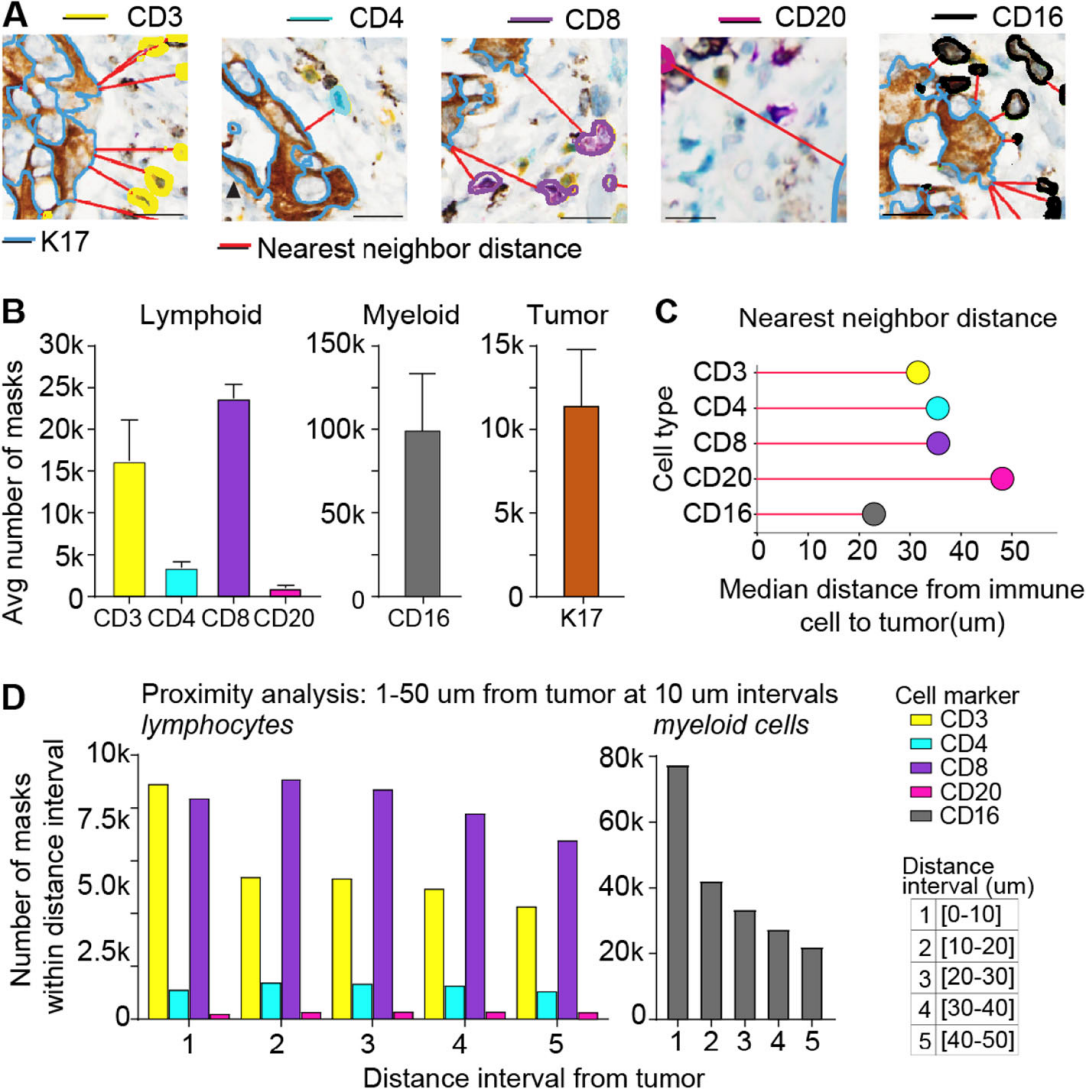
Example of analyses describing immune-tumor spatial relationships in mIHC-stained PDAC tissue. **a** Representative images of segmentation boundaries of detected tumor nests and immune cells labelled with IHC biomarkers: K17 (blue segmentation boundary), CD3 (yellow boundary), CD4 (teal boundary), CD8 (purple boundary), CD20 (magenta boundary), and CD16 (black boundary). Red lines indicate nearest neighbor distance vector connecting each immune cell to the nearest tumor nest. **b** Average number of masks per case for each of the different cell classes in three WSIs. **c** Median nearest neighbor distances for each immune cell class across cases. **d** Proximity analysis showing the number of detected masks for each cell class at 10 um distance intervals from the tumor boundary. *Note: Nearest Neighbor analyses are asymmetric and Nearest Neighbor analyses from each tumor nest to nearest immune cell are shown in supplemental Fig. 4

**https://doi.org/10.1186/s13000-020-01003-0**

Following publication of the original article [1], the authors noticed that the figures were missing from the article. The accompanying figures are presented here. The publisher apologizes to the authors and readers for the inconvenience. The original article has been updated.
