# Supplementary material for: Deep learning-based image analysis methods for brightfield-acquired multiplex immunohistochemistry images
Source: Diagn Pathol. 2020 Jul 28;15:100. doi: 10.1186/s13000-020-01003-0 (PMC7385962; doi:10.1186/s13000-020-01003-0)
Supplement: Supplementary file 1 — Additional file 1: Supplemental material. [file 13000_2020_1003_MOESM1_ESM.pdf]

Supplemental table 1. *Automated staining parameters*

	Reagent	Temperature	Time
**Baking**		60	20
**Deparaffinization**		69	8
		69	8
		69	8
**Antigen retrieval**	CC1 (high pH = 8)	95	64
	Inhibitor CM (H2O2)		12
**1**	**CD16 (SP175)**	37	24
	Anti-Rb HQ	37	12
	AntiHQ HRP		12
	DISC Ag C		16
**2**	Denaturation (CC2 low pH)	93	8
	**KDX K17**	disable heat	60
	Anti-Ms HQ	37	20
	Anti-HQ HRP		20
	ChromoMap DAB		7
**3**	Denaturation (CC2 low pH)	93	8
	**Anti-CD8 (SP57)**	disable heat	20
	Anti-Rb HQ	37	12
	Anti-HQ HRP		12
	DISC H2O2		32
**4**	Denaturation (CC2 low pH)	93	8
	**Anti-CD3 (2GV6)**	37	32
	Anti-Rb NP	37	20
	Anti-NP AP		20
	DISCO Yellow		32
**5**	Denaturation (CC2 low pH)	93	8
	**Anti-CD4 (SP35)**	37	32
	Anti-Rb HQ	37	20
	Anti-HQ HRP		20
	Teal HRP H2O2		16
	Teal HRP Act		16
**6**	Denaturation (CC2 low pH)	93	8
	**Anti-CD20**	37	20
	Anti-Ms NP	37	8
	Amp Clear		8
	Anti-NP AP		8
	DISC Red		8

Supplemental table 2. *Multiplex IHC reagent information*

Category	Reagent	Cat#
Primary antibodies	K17	
	CD3	790-4341
	CD4	790-4423
	CD8	790-4460
	CD20	760-2531
	CD16	760-4863
Hapten-conjugated secondaries	anti-ms HQ	760-4814
	anti-rb HQ	760-4815
	anti-ms NP	760-4816
	anti-rb NP	760-4817
Enzyme-conjugates	anti-HQ HRP	760-4820
	anti-NP AP	760-4827
Chromogens	ChromoMap DAB	760-159
	Discovery Yellow	760-239
	Discovery Teal	760-247
	Discovery Purple	760-229
	Discovery Red	760-228
	Discovery Silver	760-227
Bulk reagents	Discovery Wash (10x.	950-510
	Liquid Coverslip (PREDILUTE.	650-010
	Reaction Buffer (10x.	950-300
	DISCOVERY CC1 (PREDILUTE.	950-500
	Cell Conditioning 2 (Predilute; pH = 6.	950-123
	Silver Wash II (PREDILUTE.	780-003
	Amp Clear	760-4841
	Hematoxylin II	790-2208
	Bluing Reagent	760-2037

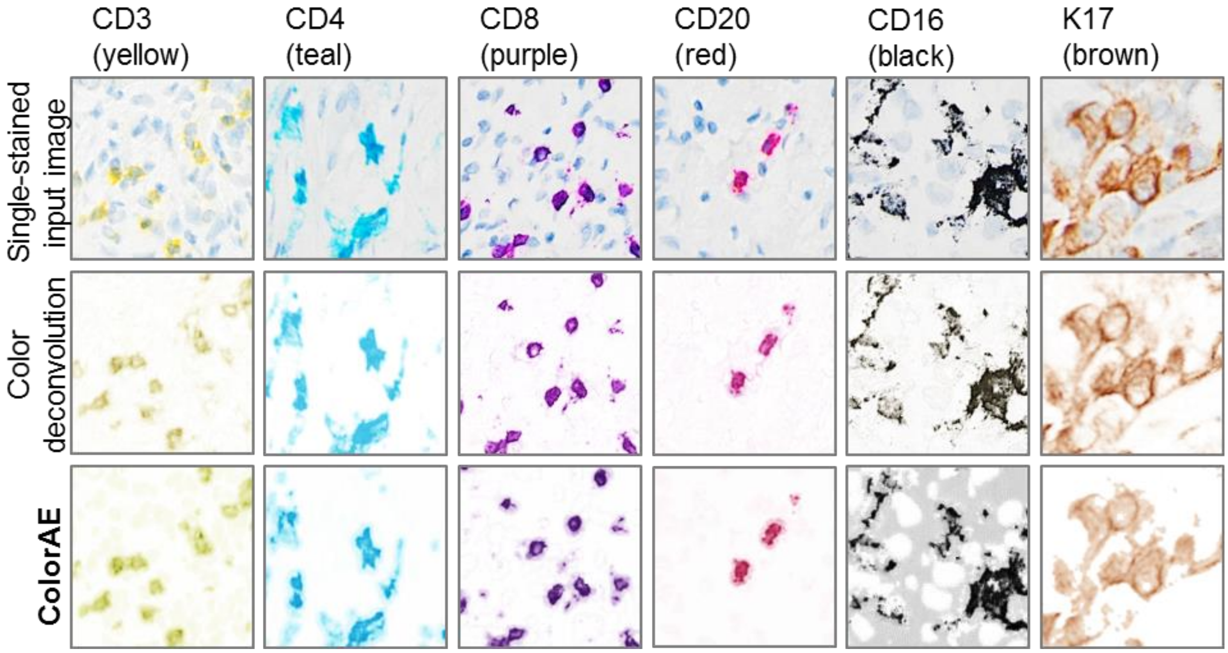

Supplemental Figure 1. Performance of ColorAE compared to "ground truth" generated with traditional color decomposition. We evaluated randomly extracted patches from single marker IHC WSIs for each cell class designated by a specific color. Traditional color decomposition was compared to ColorAE for all cell classes.

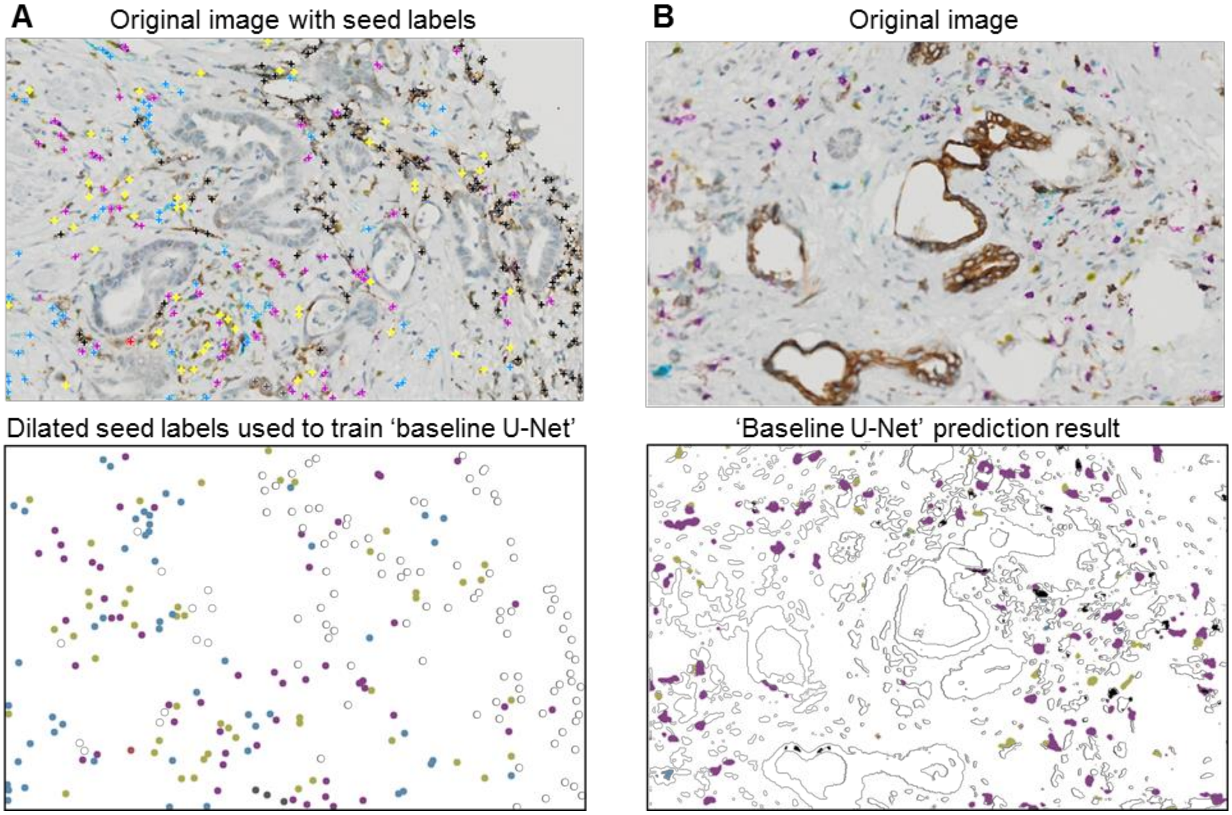

Supplemental Figure 2. Baseline U-Net training and performance. We evaluated the performance of the baseline U-NET model trained with conservatively expanded seed labels placed at the center of the cells. Since lymphocytes are circular to ovoid, this model can serve as a baseline model to evaluate the superpixel-trained U-Net model and ensemble methods. **A.** Image of a patch with seed labels overlaid (top) and the dilated seed labels generated for training the baseline 'U-NET' (bottom). **B.** Image of a patch (top) and predictions generated by the 'baseline U-NET' (bottom).

Supplemental table 3. *Comparison of 'baseline' circle-trained UNET to both superpixel-trained UNET and ensemble method*

	Model	CD3	CD4	CD8	CD20
F1	circle-trained UNET	0.280	0.619	0.323	0.218
	superpixel-trained UNET	0.628	0.661	0.628	0.353
	Union anchor AE	0.662	0.732	0.731	0.346
Recall	circle-trained UNET	0.972	0.65	0.974	0.216
	superpixel-trained UNET	0.931	0.675	0.881	0.232
	Union anchor AE	0.974	0.795	0.872	0.212
Precision	circle-trained UNET	0.171	0.669	0.201	0.62
	superpixel-trained UNET	0.473	0.647	0.488	0.736
	Union anchor AE	0.501	0.678	0.629	0.944

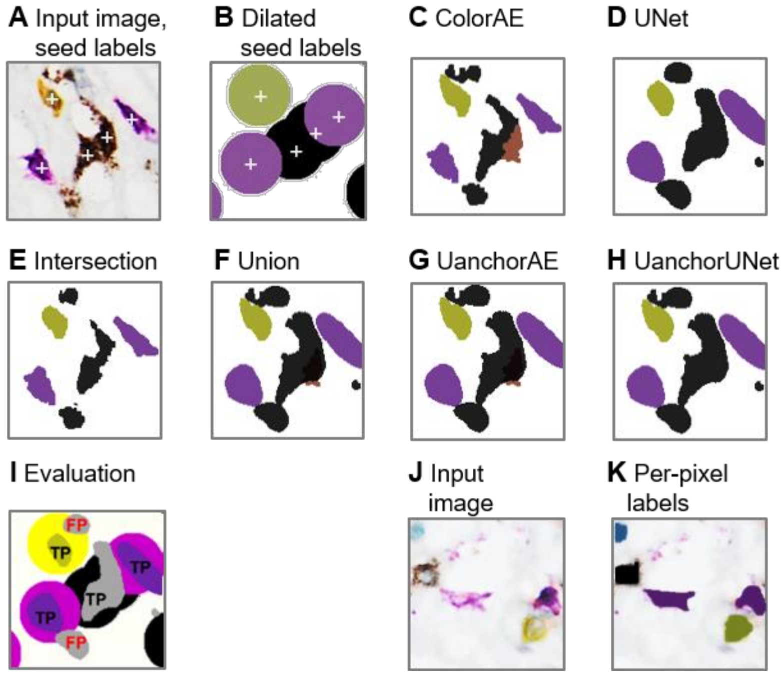

Supplemental Figure 3. Results of model performance against ground truth. ***A-H.*** *Ground truth as dilated seed labels:* **A.** Representative images of an input image with seed labels, **B.** the dilated seed labels used for evaluation, and **C-I.** the prediction masks generated by ColorAE, U-Net, and the ensemble methods: Intersection, Union, Union anchor ColorAE (UanchorAE), and Union anchor U-Net (UanchorUNet). **I.** Shows an example of how prediction masks were assessed; U-Net prediction masks are overlaid over dilated seed labels and labeled as true positive (TP, black) or false positive (FP, red). ***J-K.*** *Ground truth as hand-drawn per-pixel labels:* **J.** original input image. and **K.** per-pixel hand-drawn labels.

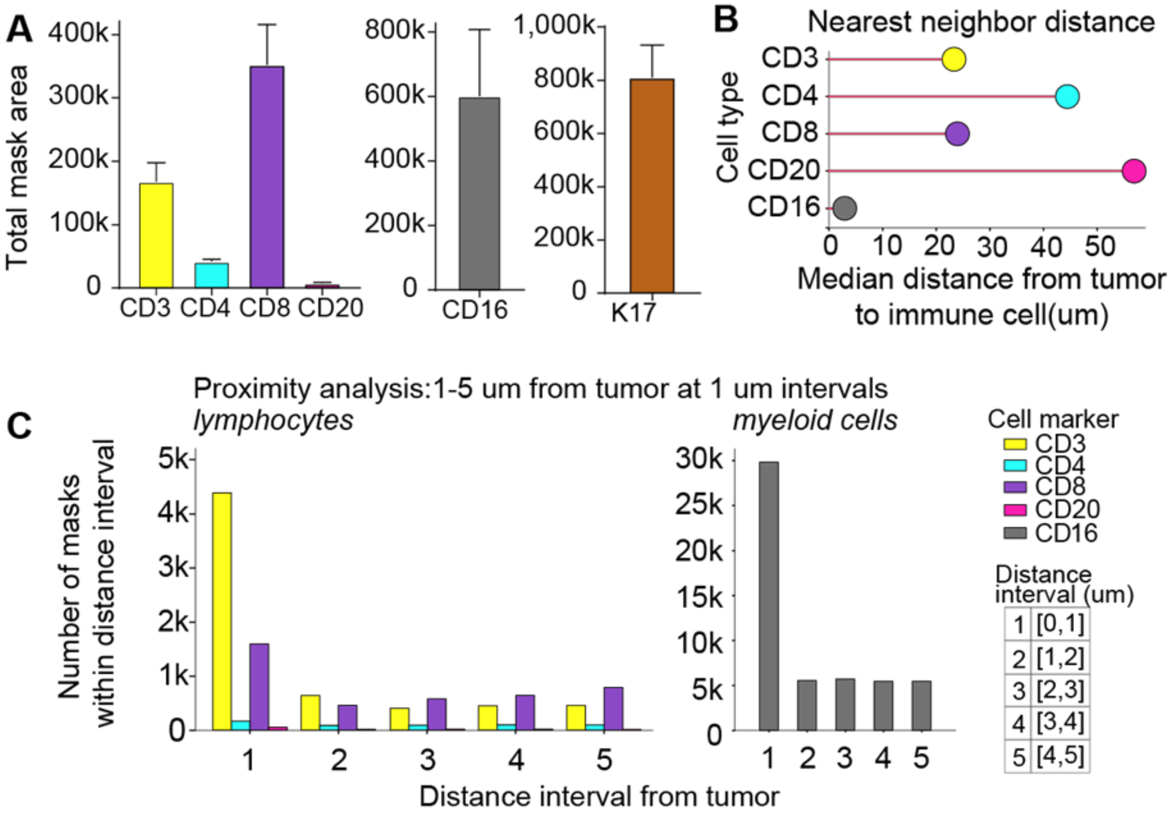

Supplemental Figure 4. mIHC spatial analysis results. A. Average total mask area (px) per case for each cell class across three WSIs. **B**. Median nearest neighbor distance from each tumor mask to the single closest immune cell of each cell class across the tumor region of three mIHC WSIs. Note this differs from results shown in Fig. 6, which counts the distance from every immune cell to the nearest tumor mask. **C**. Proximity analysis showing the number of masks for each cell class at 1 μm distance intervals from the tumor boundary.
